# Comparative mediation roles of body shape indices in the association between Healthy Eating Index-2015 and phenotypic age acceleration: A cross-sectional study based on NHANES 2005–2010

**DOI:** 10.1097/MD.0000000000050734

**Published:** 2026-09-18

**Authors:** Yingying Qian

**Affiliations:** a Department of Geriatrics, Second Affiliated Hospital, School of Medicine, Zhejiang University, Hangzhou, Zhejiang, China.

**Keywords:** body shape indices, cross-sectional study, diet quality, Healthy Eating Index-2015, mediation analysis, phenotypic age acceleration

## Abstract

Aging is a universal physiological process, yet the rate of aging varies significantly among individuals. Phenotypic Age is a biomarker-based measure that reflects biological aging by quantifying the deviation of an individual’s physiological state from their chronological age. Diet quality, as a modifiable factor, may influence the aging process; however, its association with phenotypic aging and the underlying mechanisms remain unclear. We analyzed data from 12,511 U.S. adults aged ≥ 20 years from the National Health and Nutrition Examination Survey 2005–2010. Diet quality was assessed using the Healthy Eating Index-2015 (HEI-2015), and phenotypic age acceleration was derived from 9 clinical biomarkers. Survey-weighted multivariable linear and logistic regression models were used to examine the association between HEI-2015 and phenotypic age acceleration. Mediation analysis was conducted to compare the indirect effects of 4 body shape indices: body mass index, waist-to-height ratio (WHtR), Body Roundness Index (BRI), and A Body Shape Index. Multivariable logistic regression showed that each 1-point increase in HEI-2015 was associated with lower odds of accelerated aging (odds ratio = 0.98, 95% confidence interval: 0.97–0.98, *P* < .001). All 4 body shape indices exhibited partial mediation effects, with WHtR and BRI showing the largest mediation proportions (29.23% and 28.90%, respectively). Subgroup and sensitivity analyses yielded consistent results, supporting the robustness of the findings. Higher diet quality is associated with lower odds of biological age acceleration, partially mediated through improved body shape, particularly indicators of central adiposity such as WHtR and BRI. These findings support a dual-targeted strategy integrating dietary improvement and body shape management to more precisely mitigate aging acceleration.

## 1. Introduction

Aging has emerged as a central issue in global public health. As populations age and life expectancy increases, many countries face growing challenges related to age-associated diseases and functional decline.^[1]^ While aging is a natural physiological process, it has been consistently linked to elevated risks of chronic conditions and reduced healthspan.^[2]^ Importantly, the pace of aging varies substantially across individuals, reflecting marked biological heterogeneity that cannot be fully explained by chronological age alone.^[3]^

Phenotypic age, a composite biomarker-based metric, has been proposed to better capture biological aging.^[4]^ By integrating multiple clinical and biochemical markers, phenotypic age has demonstrated stronger predictive ability for chronic disease incidence and all-cause mortality than chronological age.^[5]^ As such, phenotypic age acceleration (PhenoAgeAccel), defined as the discrepancy between phenotypic and chronological age, has been increasingly used to capture interindividual differences in biological aging.

Diet quality, as a modifiable lifestyle factor, plays a key role in shaping biological aging trajectories.^[6]^ Prior studies have shown that healthy dietary patterns, such as the Mediterranean and DASH diets, are associated with lower risks of chronic diseases and improved longevity.^[7,8]^ The Healthy Eating Index-2015 (HEI-2015), developed to reflect adherence to the Dietary Guidelines for Americans, provides a comprehensive assessment of overall diet quality across multiple dietary components.^[9,10]^ Compared with single-nutrient or pattern-based indices, HEI-2015 captures both adequacy and moderation dimensions of dietary intake, making it particularly suitable for population-based aging research. Although recent studies have begun to explore the link between diet and aging, including the potential mediating role of body mass index (BMI),^[11]^ evidence specifically linking HEI-2015 to PhenoAgeAccel remains limited.^[12]^ However, the underlying mechanisms linking diet quality to biological aging remain poorly understood, and it is yet to be determined whether alternative body shape indices that more accurately reflect visceral adiposity offer superior explanatory power over BMI in these pathways.

Body shape indices, including BMI, waist-to-height ratio (WHtR), Body Roundness Index (BRI), and A Body Shape Index (ABSI), reflect different aspects of adiposity and fat distribution that are closely related to metabolic dysfunction and aging-related health risks.^[13]^ Unfavorable body shape profiles have been associated with chronic low-grade inflammation, insulin resistance, and mitochondrial dysfunction, all of which may contribute to accelerated biological aging.^[14,15]^ From a biological perspective, diet quality may influence these indices by modulating metabolic health and systemic inflammation, which in turn act as drivers of cellular senescence. Given the strong impact of dietary habits on body composition, body shape indices may represent key intermediate pathways linking diet quality to biological aging. However, the relative contributions of different body shape indices in this pathway remain insufficiently characterized.

Therefore, the present study aimed to examine the association between diet quality, as assessed by HEI-2015, and PhenoAgeAccel, and compare the potential mediating roles of BMI, WHtR, BRI, and ABSI in this association. By clarifying the relationships among diet quality, body shape characteristics, and biological aging, this study may provide etiological insights into modifiable factors related to healthy aging.

## 2. Methods

### 2.1. Study population

This study utilized data from the National Health and Nutrition Examination Survey (NHANES), an ongoing, nationally representative, cross-sectional survey conducted by the U.S. Centers for Disease Control and Prevention. The 2005–2010 cycles were selected because laboratory biomarkers required for phenotypic age calculation were available during these periods. All participants provided written informed consent, and the study protocols were approved by the National Center for Health Statistics Ethics Review Board. This study was conducted and reported in accordance with the Strengthening the Reporting of Observational Studies in Epidemiology guidelines for cross-sectional studies.

**Figure 1.**
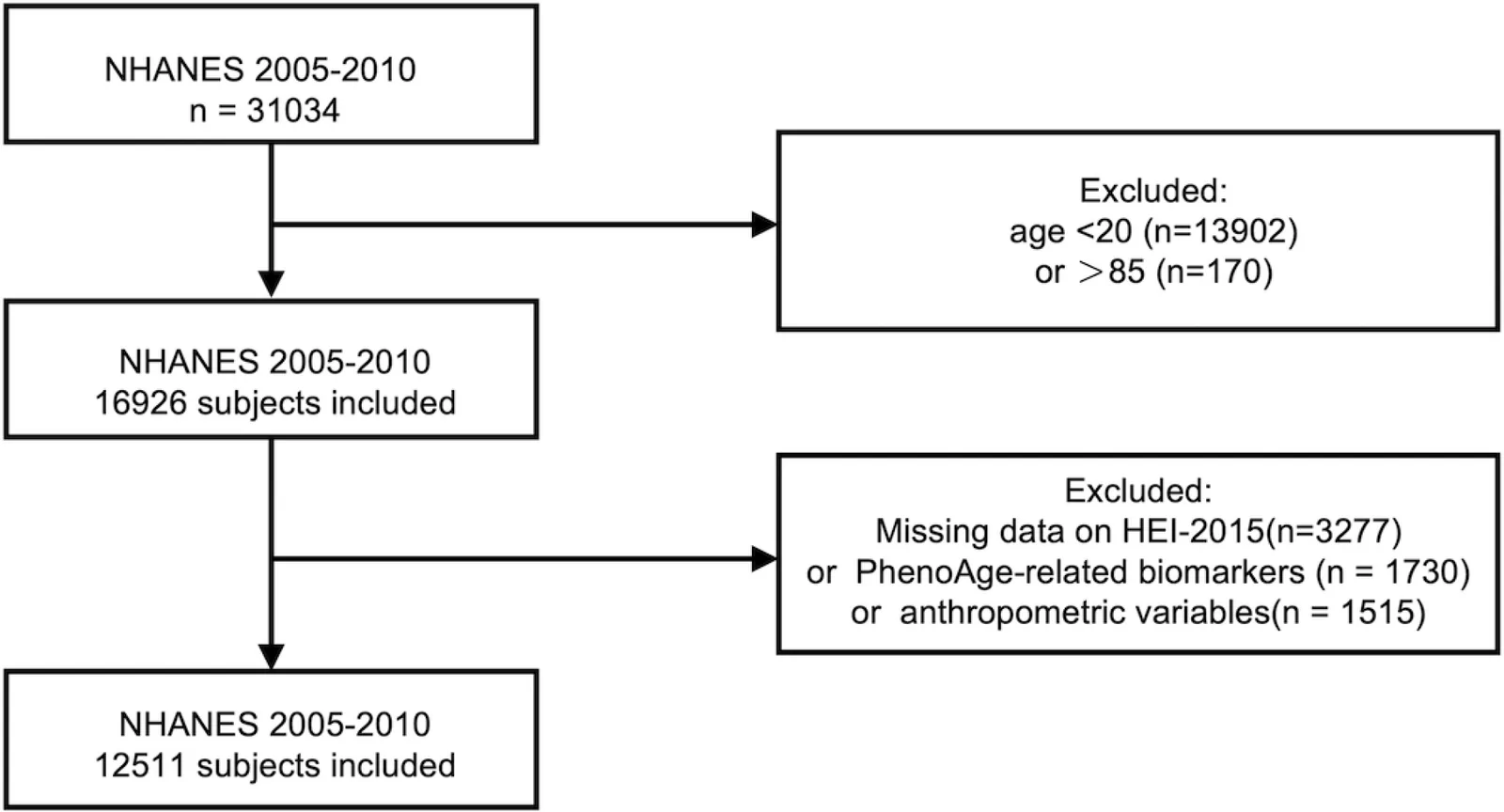
Flowchart of participant selection from NHANES 2005–2010. HEI-2015 = Healthy Eating Index-2015, NHANES = National Health and Nutrition Examination Survey.

A total of 31,034 participants were initially enrolled across the 3 survey cycles. We excluded individuals aged < 20 years (n = 13,902) and those aged > 85 years (n = 170) because age is top-coded at 85 years in NHANES, precluding accurate estimation of PhenoAgeAccel. Participants with missing data on HEI-2015 (n = 3277), phenotypic age biomarkers (n = 1730), or body shape indices (n = 1515) were further excluded. Missing covariate data were handled using multiple imputation by chained equations. Missing data rates for each covariate are presented in Table S1, Supplemental Digital Content 1. Five imputed datasets were generated, and estimates were combined using Rubin rules. The final analytical sample consisted of 12,511 adults aged 20 to 85 years (Fig. 1).

### 2.2. Diet quality assessment

Dietary intake was assessed using 2 nonconsecutive 24-hour dietary recall interviews conducted by trained personnel. Diet quality was evaluated using the HEI-2015, which reflects adherence to the 2015–2020 Dietary Guidelines for Americans. HEI-2015 scores were calculated based on the average intake across the 2 dietary recalls.

The HEI-2015 comprises 13 dietary components, including 9 adequacy components (total fruits, whole fruits, total vegetables, greens and beans, whole grains, dairy, total protein foods, seafood and plant proteins, and fatty acids) and 4 moderation components (refined grains, sodium, added sugars, and saturated fats). Component scores were calculated using a density-based approach, with intakes expressed per 1000 kcal for most components, except for fatty acids, added sugars, and saturated fats, which were expressed as percentages of energy. For adequacy components, higher intakes received higher scores; for moderation components, lower intakes received higher scores (reverse scoring). Intakes between the minimum and maximum standards were scored proportionately using linear interpolation. The component scores were summed to obtain the total HEI-2015 score for each participant. HEI-2015 component and total scores were calculated at the individual level. The total score ranges from 0 to 100, with higher scores indicating better overall diet quality. In the present study, HEI-2015 was primarily treated as a continuous variable in the analyses.

### 2.3. Phenotypic age and accelerated aging

Phenotypic age was calculated using the method proposed by Levine et al^[16]^, integrating 9 biomarkers (albumin, creatinine, glucose, C-reactive protein, lymphocyte percentage, red cell distribution width, mean cell volume, alkaline phosphatase, and white blood cell count) along with chronological age, as originally specified.

PhenoAgeAccel was defined as the residual from regressing phenotypic age on chronological age.^[17]^ A positive value indicates accelerated aging, while a negative value reflects decelerated aging.^[18]^

Phenotypic age is determined using the following formula:


$$\begin{array}{c} Phenotypic\;Age=141.50+\frac{\ln\left[-0.00553\times \ln\left(1-M\right)\right]}{0.09165} \end{array}$$

Where:


$$\begin{array}{c} M=1-\exp\left(\frac{-1.51714\times \exp\left(xb\right)}{0.0076927}\right) \end{array}$$

and:


$$\begin{array}{c} \begin{array}{cc} xb=-19.907-0.0336\times Albumin+0.0095\\ & \times Creatinine\;+0.1953\times Glucose\\ & +0.0954\times \ln\left(CRP\right)-0.0120\times Lymphocyte\;Percent\\ & +0.0268\times Mean\;Cell\;Volume+0.3306\\ & \times Red\;Cell\;Distribution\;Width\\ & +0.00188\times Alkaline\;Phosphatase+0.0554\\ & \times White\;Blood\;Cell\;Count\\ & +0.0804\times Chronological\;Age \end{array} \end{array}$$

### 2.4. Body shape indices

Anthropometric measurements, including height, weight, and waist circumference, were performed by trained health technicians in the Mobile Examination Center using standardized equipment according to standardized NHANES protocols. Four indices were used to characterize body shape and fat distribution, capturing different aspects of overall and central adiposity. All body shape indices were treated as continuous variables in the analyses.


$$\begin{array}{c} Body\;Mass\;Index\;(BMI)=weight\;(kg)/height^{2}(m^{2}); \end{array}$$

$$\begin{array}{c} \begin{array}{cc} Waist\text{-}to\text{-}Height\;Ratio\left(WHtR\right)\\ & =waist\;circumference\left(cm\right)/height\left(cm\right); \end{array} \end{array}$$

$$\begin{array}{c} \begin{array}{cc} Body\;Roundness\;Index\;\left(BRI\right)=364.2-365.5\\ & \times \surd {\left[1-(waist/\left(\pi \times height\right)\right)}^{2}]; \end{array} \end{array}$$

$$\begin{array}{cc} A\;Body\;Shape\;Index\left(ABSI\right)\\ & =waist\;circumference/\left(BMI^{\left(2/3\right)}\times height^{\left(1/2\right)}\right). \end{array}$$

### 2.5. Covariates

Covariates were selected a priori based on previous literature and biological plausibility and were categorized as demographic, lifestyle, and health-related factors. Demographic variables included sex, age, race/ethnicity, education level, marital status, and family income, assessed using the poverty-income ratio. Lifestyle factors included smoking status, alcohol consumption, and total energy intake. Health-related covariates included self-reported physician-diagnosed chronic conditions, including diabetes, hypertension, and cancer (yes/no). Given that fasting glucose is an integral component of the phenotypic age calculation, diabetes status was not included as a covariate in the primary models to minimize potential overadjustment. Sensitivity analyses additionally adjusting for diabetes were conducted to assess the robustness of the findings. Detailed variable definitions and data collection procedures are available on the NHANES website (https://www.cdc.gov/nchs/nhanes/).

### 2.6. Statistical analysis

All analyses accounted for the complex multistage sampling design of NHANES, incorporating survey weights, strata, and primary sampling units. Continuous variables were summarized as weighted means with standard errors, and categorical variables as weighted percentages. Group differences were evaluated using survey-weighted *t*-tests for continuous variables and Rao–Scott chi-square tests for categorical variables.

Survey-weighted multivariable linear regression models were used to examine the association between HEI-2015 and PhenoAgeAccel as a continuous outcome. In secondary analyses, PhenoAgeAccel was dichotomized to define accelerated aging (PhenoAgeAccel > 0), and survey-weighted logistic regression models were applied to estimate odds ratios (ORs) and 95% confidence intervals (CIs). To address potential nonlinearity, restricted cubic spline regression was utilized with 3 knots placed at prespecified percentiles of the weighted HEI-2015 distribution. In addition, segmented (two-piecewise linear) regression models were applied to formally test for threshold effects, and log-likelihood ratio tests were used to compare the segmented models with standard linear models. Three sequential models were constructed: Model 1, unadjusted; Model 2, adjusted for age, sex, race/ethnicity, education, income, and marital status; and Model 3, further adjusted for smoking, alcohol use, cancer, hypertension, and total energy intake.

Mediation analyses were conducted using a counterfactual-based framework to decompose the total effect into natural direct and indirect effects to evaluate the indirect effects of body shape indices (BMI, WHtR, BRI, and ABSI) on the association between HEI-2015 and PhenoAgeAccel. For each mediator, a linear regression model was fitted for the mediator and a linear regression model for the outcome, adjusting for the same covariates as in Model 3. Indirect effects and 95% CIs were estimated using nonparametric bootstrap resampling with 1000 iterations.

Sensitivity analyses were performed by additionally adjusting for self-reported physician-diagnosed diabetes, hypertension, and cancer to assess the robustness of the findings. Specifically, considering that fasting glucose is a component of phenotypic age, models excluding diabetes status were compared to evaluate potential overadjustment. All statistical analyses were conducted using R software (version 4.3.1), and statistical significance was defined as a two-sided *P* value < .05.

## 3. Results

### 3.1. Characteristics of study population

A total of 12,511 participants were included in the final analysis, with 2022 (13%) classified into the accelerated aging group (PhenoAgeAccel ≥ 0) and 10,489 (87%) into the delayed aging group (PhenoAgeAccel < 0). Compared to the delayed aging group, individuals with accelerated aging were significantly older (median 50 vs 46 years) and more likely to be Non-Hispanic Black (18% vs 9.3%). They also exhibited a substantially higher prevalence of chronic conditions, including hypertension (47% vs 28%), diabetes (23% vs 5.6%), and a history of cancer (14% vs 8.5%; all *P* < .001).

Regarding adiposity markers, those with accelerated aging demonstrated significantly higher levels across all 4 indices, with a median BMI of 31 kg/m^2^ compared to 27 kg/m^2^ in the delayed aging group (*P* < .001). Furthermore, diet quality was notably poorer in the accelerated aging group, as indicated by lower median HEI-2015 scores (48 vs 51, *P* < .001; Table 1). Detailed comparisons of individual HEI-2015 component scores between the 2 groups are presented in Table S5, Supplemental Digital Content 2.

**Table 1:** Baseline characteristics of participants stratified by phenotypic age acceleration status.

Characteristic	Delayed N = 10,489 (87%)	Accelerated N = 2022 (13%)	*P*-value
Chronological age	46 (33 to 58)	50 (38 to 64)	<.001
Phenotypic age	37 (25 to 50)	55 (41 to 71)	<.001
PhenoAgeAccel	−8.3 (−11.6 to −5.2)	3.3 (1.3 to 6.6)	<.001
Gender			.3
Male	217,699,713 (47%)	30,553,242 (46%)	
Female	242,032,823 (53%)	36,496,884 (54%)	
Race and ethnicity			<.001
Mexican American	36,381,637 (7.9%)	5,497,962 (8.2%)	
Other Hispanic	19,045,708 (4.1%)	3,195,927 (4.8%)	
Non-Hispanic White	337,102,790 (73%)	44,266,178 (66%)	
Non-Hispanic Black	42,710,822 (9.3%)	11,737,366 (18%)	
Other/multiracial	24,491,578 (5.3%)	2,352,693 (3.5%)	
Educational level			<.001
<9th grade	23,250,465 (5.1%)	5,648,463 (8.4%)	
9–11th grade	50,020,389 (11%)	12,147,794 (18%)	
High school or GED	108,308,381 (24%)	19,588,468 (29%)	
Some college/AA degree	142,349,477 (31%)	19,125,208 (29%)	
College or above	135,803,823 (30%)	10,540,193 (16%)	
Family income-poverty level			<.001
<1.0	53,467,270 (12%)	13,036,343 (19%)	
1–3.0	157,941,507 (34%)	27,730,267 (41%)	
>3.0	248,323,759 (54%)	26,283,516 (39%)	
Marital status			<.001
Married	271,856,686 (59%)	33,518,328 (50%)	
Divorced	44,766,261 (9.7%)	9,963,076 (15%)	
Separated	9,524,607 (2.1%)	2,646,680 (3.9%)	
Other*	133,584,982 (29%)	20,922,042 (31%)	
Alcohol drinking			<.001
<12drinks/yr	106,450,985 (23%)	20,107,338 (30%)	
≥12drinks/yr	353,281,551 (77%)	46,942,787 (70%)	
Smoking status			<.001
Never smoker	251,621,490 (55%)	27,077,267 (40%)	
Smoker	208,111,046 (45%)	39,972,859 (60%)	
Cancer history			<.001
No	420,722,857 (92%)	57,930,536 (86%)	
Yes	39,009,679 (8.5%)	9,119,590 (14%)	
Diabetes			<.001
No	426,840,222 (93%)	49,938,958 (74%)	
Near	7,336,565 (1.6%)	1,426,076 (2.1%)	
Yes	25,555,750 (5.6%)	15,685,092 (23%)	
Hypertension			<.001
No	331,070,741 (72%)	35,527,643 (53%)	
Yes	128,661,795 (28%)	31,522,483 (47%)	
BMI	27 (24 to 31)	31 (26 to 38)	<.001
BRI	4.67 (3.53 to 6.04)	6.33 (4.74 to 8.38)	<.001
WHtR	0.57 (0.51 to 0.63)	0.64 (0.57 to 0.72)	<.001
ABSI	0.081 (0.078 to 0.084)	0.083 (0.079 to 0.086)	<.001
Total calories (kcal)	1983 (1535 to 2570)	1888 (1417 to 2389)	<.001
HEI2015 score	51 (43 to 60)	48 (41 to 56)	<.001

Values are presented as weighted percentages for categorical variables and weighted medians (interquartile ranges) for continuous variables. Phenotypic age acceleration (PhenoAgeAccel) was calculated as the residual from regressing phenotypic age on chronological age. Participants with PhenoAgeAccel ≥ 0 were classified as having accelerated aging, whereas those with PhenoAgeAccel < 0 were classified as having delayed aging.

ABSI = A Body Shape Index, BMI = body mass index, BRI = Body Roundness Index, GED = General Educational Development, HEI-2015 = Healthy Eating Index-2015, PhenoAgeAccel = phenotypic age acceleration, WHtR = waist-to-height ratio.

* Represents widowed, never married, and living with partner.

### 3.2. Association between HEI-2015 and accelerated aging

Multivariable logistic regression models demonstrated a consistent inverse association between diet quality and the odds of accelerated aging. When treated as a continuous variable, each 1-unit increase in HEI-2015 score was associated with a 2% reduction in the odds of accelerated aging in the fully adjusted Model 3 (OR = 0.98; 95% CI: 0.98–0.99; *P* < .001).

When HEI-2015 was analyzed by quartiles, a clear dose-response relationship was observed across all models. In the most comprehensive model (Model 3), which accounted for demographic factors, lifestyle, and comorbidities, participants in the highest quartile (Q4) had a 33% lower likelihood of accelerated aging compared to those in the lowest quartile (Q1; OR = 0.67; 95% CI: 0.57–0.77; *P* < .001). The intermediate quartiles also showed a progressive decrease in odds, with Q3 exhibiting a significant 19% reduction (OR = 0.81; 95% CI: 0.70–0.93; *P* = .003; Table 2).

**Table 2:** Association between HEI-2015 and phenotypic age acceleration in U.S. adults.

HEI-2015	Model1		Model2		Model3	
	OR (95% CI)	*P*	OR (95% CI)	*P*	OR (95% CI)	*P*
Continuous	0.98 (0.98–0.99)	**<.001**	0.99 (0.98–0.99)	**<.001**	0.98 (0.98–0.99)	**<.001**
Quartiles						
Q1	1.00 (Reference)		1.00 (Reference)		1.00 (Reference)	
Q2	0.89 (0.79–1.02)	.084	0.95 (0.84–1.09)	.466	0.92 (0.80–1.05)	.206
Q3	0.77 (0.67–0.88)	**<.001**	0.87 (0.76–0.99)	**.048**	0.81 (0.70–0.93)	**.003**
Q4	0.62 (0.54–0.71)	**<.001**	0.76 (0.66–0.88)	**<.001**	0.67 (0.57–0.77)	**<.001**

Odds ratios (ORs) and 95% confidence intervals (CIs) were estimated using multivariable logistic regression. Accelerated aging was defined as PhenoAgeAccel > 0. Model 1: Crude. Model 2: Adjusted for age, sex, race/ethnicity, education, income, and marital status. Model 3: Additionally adjusted for smoking, alcohol use, cancer, hypertension, and total energy intake.

Bold values indicate statistical significance at *P* < .05.

HEI-2015 = Healthy Eating Index-2015.

### 3.3. Dose–response and subgroup analyses

**Figure 2.**
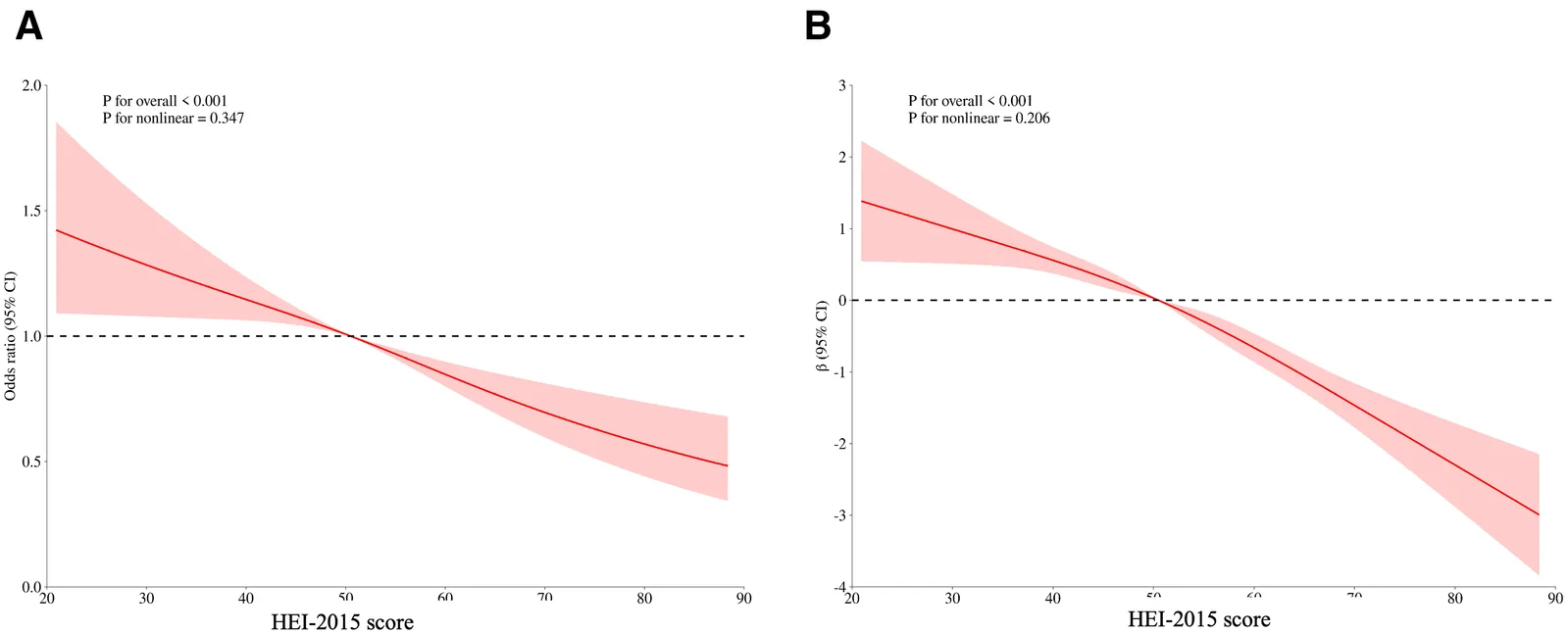
Dose–response associations between HEI-2015 and phenotypic age acceleration modeled using restricted cubic splines. (A) Association between HEI-2015 scores and the odds of phenotypic age acceleration (binary outcome), modeled using logistic regression. (B) Association between HEI-2015 scores and phenotypic age acceleration (continuous outcome), modeled using linear regression. All models were adjusted for age, sex, race/ethnicity, education level, income, and marital status, as well as smoking status, alcohol use, history of cancer, hypertension, and total energy intake. Solid lines represent the estimated associations, and shaded areas indicate 95% confidence intervals. CI = confidence interval, HEI-2015 = Healthy Eating Index-2015, OR = odds ratio.

Restricted cubic spline analysis demonstrated a significant linear inverse dose–response relationship between HEI-2015 and PhenoAgeAccel (overall *P* < .001, Fig. 2A). No evidence of nonlinearity was found (*P* for nonlinearity > .05), suggesting that the protective association of higher diet quality against accelerated biological aging remains stable across the entire range of HEI-2015 scores. Segmented regression analysis also showed no evidence of a threshold effect (*P* for likelihood ratio test = .490; Table S3, Supplemental Digital Content 3), further supporting an approximately linear association.

To assess the robustness of the findings, a sensitivity analysis was conducted using PhenoAgeAccel as a continuous variable under the fully adjusted model. The results similarly revealed a significant linear inverse association (Fig. 2B), further supporting the reliability of the main analysis. Multivariable linear regression results for the association between HEI-2015 and PhenoAgeAccel are detailed in Table S2, Supplemental Digital Content 4.

**Figure 3.**
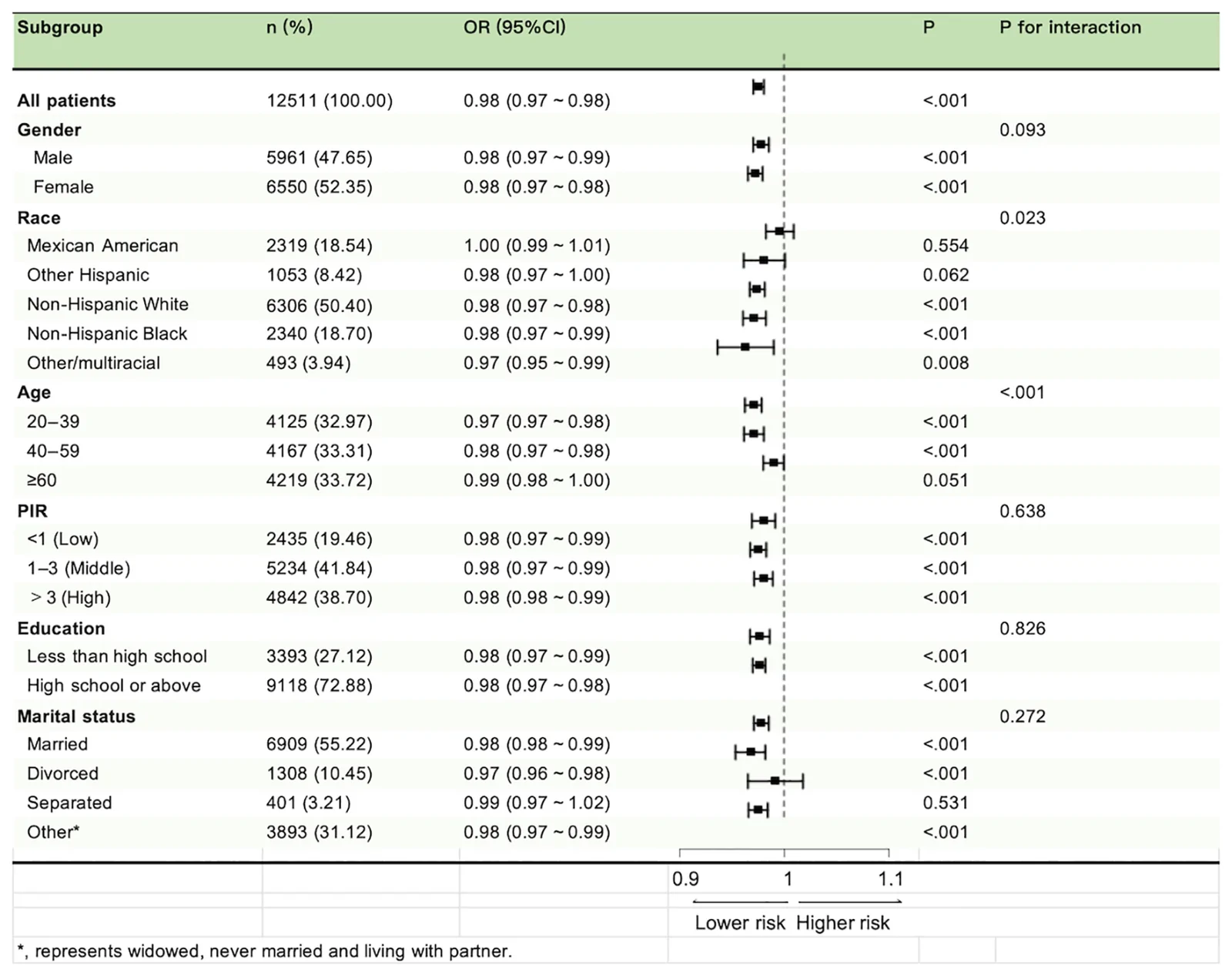
Forest plot of subgroup analyses for the association between HEI-2015 and phenotypic age acceleration. Multivariable logistic regression models were used to estimate odds ratios (ORs) and 95% confidence intervals (CIs) for the association between HEI-2015 and phenotypic age acceleration across subgroups. All models were adjusted for smoking status, alcohol use, history of cancer, hypertension, and total energy intake. HEI-2015 = Healthy Eating Index-2015, PIR = poverty–income ratio.

Stratified analyses demonstrated that the inverse association between HEI-2015 and the odds of accelerated aging remained robust across most demographic and lifestyle subgroups (Fig. 3). No significant interactions were observed for sex, education, income, or marital status (all *P* for interaction > .05). However, age significantly modified the association (*P* for interaction < .001): the inverse relationship was most pronounced in younger adults (20–39 years: OR = 0.97; 95% CI: 0.97–0.98) and became attenuated in the oldest group (≥ 60 years: OR = 0.99; 95% CI: 0.98–1.00).

Regarding race and ethnicity, a significant inverse association was observed across all groups, including Non-Hispanic White (OR = 0.98; 95% CI: 0.97–0.99), Non-Hispanic Black (OR = 0.98; 95% CI: 0.97–0.99), and Mexican American participants (OR = 0.98; 95% CI: 0.97–0.99; Fig. 3). These findings indicate that higher diet quality is universally associated with reduced odds of accelerated aging across diverse ethnic backgrounds.

### 3.4. Mediating role of body shape indices

Multivariable linear regression models confirmed that higher HEI-2015 scores were significantly associated with lower levels of all 4 body shape indices, including BMI, WHtR, BRI, and ABSI (all *P* < .001). Simultaneously, each of these indices exhibited a significant positive association with PhenoAgeAccel (all *P* < .001), suggesting that unfavorable body shape profiles are robust predictors of accelerated biological aging (Table S4, Supplemental Digital Content 5).

Mediation analysis further revealed that all 4 body shape indices served as significant partial mediators in the association between diet quality and PhenoAgeAccel (Table 3). Notably, the mediation proportions varied across different metrics, reflecting their distinct capacities to capture the biological link between nutrition and aging. WHtR and BRI demonstrated the most substantial mediating effects, accounting for 29.23% and 28.90% of the total effect, respectively. In comparison, BMI exhibited a moderate mediation proportion of 22.32%, while ABSI contributed the least (7.62%).

**Table 3:** Adiposity index mediating the association between HEI-2015 and phenotypic age acceleration.

Mediator	Total effect (95% CI)	*P* value	ACME (95% CI)	*P* value	ADE (95% CI)	*P* value	Proportion mediated
BMI	−0.06 (−0.08 to −0.05)	<.001	−0.01 (−0.02 to −0.01)	<.001	−0.05 (−0.06 to −0.04)	<.001	22.32%
BRI	−0.06 (−0.08 to −0.05)	<.001	−0.02 (−0.02 to −0.02)	<.001	−0.05 (−0.06 to −0.04)	<.001	28.90%
ABSI	−0.06 (−0.08 to −0.05)	<.001	−0.00 (−0.01 to −0.00)	<.001	−0.05 (−0.06 to −0.04)	<.001	7.62%
WHtR	−0.06 (−0.08 to −0.05)	<.001	−0.02 (−0.02 to −0.02)	<.001	−0.05 (−0.06 to −0.04)	<.001	29.23%

Estimates reflect the effect of HEI-2015 (continuous) on phenotypic age acceleration, mediated through individual adiposity indices. Proportion mediated is calculated as ACME/Total effect. All models adjusted for covariates including age, sex, race/ethnicity, education, income, marital status, smoking, alcohol, comorbidities, and total energy intake.

ABSI = A Body Shape Index, ACME = average causal mediation effect, ADE = average direct effect, BMI = body mass index, BRI = Body Roundness Index, HEI-2015 = Healthy Eating Index-2015, WHtR = waist-to-height ratio.

**Figure 4.**
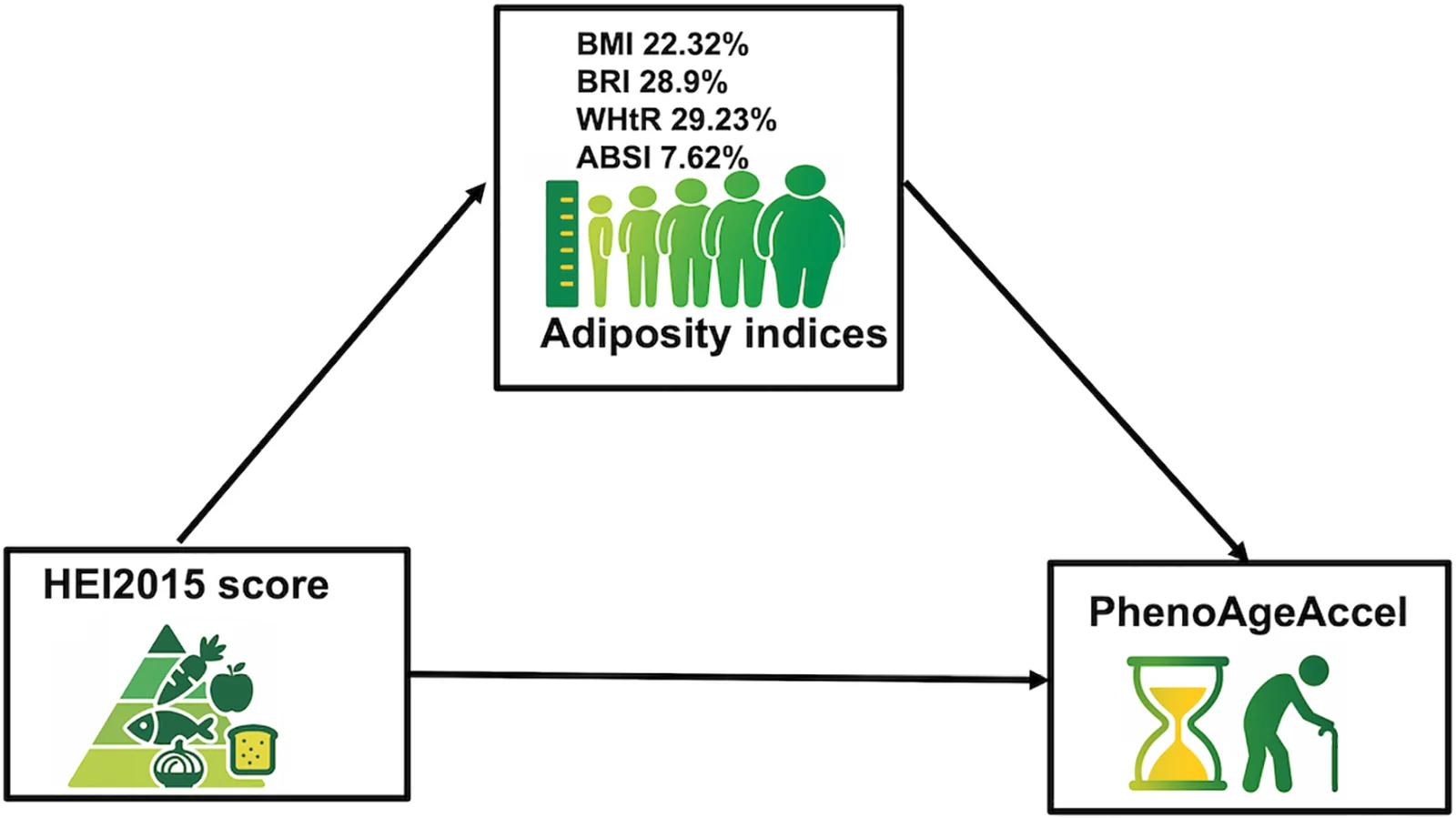
Mediation model. Mediation analysis of the association between HEI-2015 and phenotypic age acceleration through 4 body shape indices: BMI, waist-to-height ratio (WHtR), Body Roundness Index (BRI), and A Body Shape Index (ABSI). The analysis was conducted using a counterfactual-based approach with 1000 bootstrap iterations to estimate 95% confidence intervals. All models were adjusted for age, sex, race/ethnicity, education level, income, marital status, smoking status, alcohol use, history of cancer, hypertension, and total energy intake. BMI = body mass index, HEI-2015 = Healthy Eating Index-2015.

These findings are visually summarized in the conceptual mediation model (Fig. 4). The results collectively support the hypothesized “diet–body shape–aging” pathway, suggesting that the protective effect of a high-quality diet on biological aging is significantly channeled through the optimization of body composition, particularly in terms of central adiposity.

## 4. Discussion

This study, based on a nationally representative sample of U.S. adults, demonstrates that higher diet quality, as measured by the HEI-2015, is robustly associated with reduced odds of PhenoAgeAccel. Notably, our systematic comparison of multiple body shape indices reveals that central adiposity markers, specifically the WHtR and BRI, exhibit substantially stronger mediating effects (29.23% and 28.90%, respectively) compared to the traditional BMI (22.32%). These findings suggest that the protective influence of healthy dietary patterns on biological aging is significantly channeled through the optimization of body composition and fat distribution. Consequently, these results highlight the potential clinical and public health value of integrating dietary guidance with targeted management of central adiposity to mitigate aging-related health risks.

Our results align with previous research indicating that healthy dietary patterns, such as the Mediterranean and DASH diets, are linked to better health outcomes and a slower biological aging process.^[6,8,19]^ Although some studies have explored the relationship between dietary patterns and aging biomarkers,^[20]^ this study extends current knowledge by identifying a “diet–body shape–aging” mediation pathway and by highlighting the differential roles of specific body shape indices. The stronger mediating effects observed for WHtR and BRI, compared with BMI and ABSI, likely reflect fundamental differences in what these indices capture. BMI serves as a crude indicator of overall body mass and does not distinguish between lean mass and adipose tissue, whereas WHtR and BRI are specifically designed to reflect abdominal adiposity and visceral fat accumulation.

From a biological perspective, central fat accumulation is closely linked to several aging-related mechanisms: visceral adipose tissue functions as an active endocrine organ rather than a passive energy reservoir, secreting pro-inflammatory cytokines such as TNF-α and IL-6. These mediators promote chronic systemic low-grade inflammation, commonly referred to as “inflammaging,” which directly influences key components of the PhenoAge algorithm, particularly C-reactive protein and albumin levels.^[21]^ In addition, central fat accumulation is a major contributor to metabolic dysregulation by exacerbating insulin resistance and dyslipidemia, alterations that are reflected in glucose- and lipid-related parameters included in biological aging scores.^[22]^ At the cellular level, excessive lipid deposition in non-adipose tissues may impair mitochondrial biogenesis and suppress antioxidant defenses. This lipotoxic environment can trigger cellular senescence and tissue degeneration, thereby accelerating biological aging.^[23–25]^ Together, these mechanisms help explain why WHtR and BRI appear to be more sensitive indicators of phenotypic aging than BMI, and support their potential relevance as modifiable targets within dietary strategies aimed at healthy aging.

Notably, although the inverse association between HEI-2015 and PhenoAgeAccel was generally consistent across most subgroups, significant effect modification was observed by age (*P* interaction < .001) and race/ethnicity (*P* interaction = .039). The association was most pronounced among younger adults (aged 20–39 years; OR = 0.965, 95% CI: 0.952–0.978) and showed a tendency toward attenuation with increasing age. This age-related gradient may reflect greater biological plasticity in earlier adulthood, during which healthy dietary patterns could more effectively limit the accumulation of aging-related molecular damage. This observation is consistent with a life-course perspective, highlighting early adulthood as a potentially important period for nutritional strategies aimed at promoting healthy aging.^[26]^

With respect to race and ethnicity, higher diet quality was significantly associated with lower odds of PhenoAgeAccel across all examined groups (all *P* < .05). Although a statistically significant interaction was detected, the magnitude of the associations was highly comparable across racial and ethnic categories (ORs ranging from 0.974 to 0.984), suggesting that the protective association of diet quality with biological aging is largely consistent across populations. The observed interaction may reflect subtle contextual differences rather than substantial heterogeneity in biological response. Consequently, these findings support the implementation of universal, population-wide nutritional strategies to promote biological longevity across diverse demographic landscapes.

From a public health perspective, our findings underscore the importance of dietary intervention in the prevention of biological aging. First, the healthy dietary pattern captured by the HEI-2015 is practical and feasible for population-wide implementation, and related interventions such as nutrition education and dietary counseling can be readily integrated into community and primary care settings.^[9]^ Second, simple and accessible body shape indices, including WHtR and BRI, demonstrated meaningful mediating effects, suggesting that early identification of aging risk through routine anthropometric assessment may be achievable in real-world settings. Third, the stronger associations observed among younger adults suggest that dietary interventions initiated earlier in life may generate greater cumulative benefits over the life course, supporting public health strategies that emphasize nutrition education and lifestyle management from early adulthood onward.

This study has several strengths, including the use of a nationally representative large sample, a validated measure of PhenoAgeAccel, and robust statistical modeling. Importantly, our comparison of the mediation effects of multiple body shape indices offers a novel perspective on the pathways linking diet and biological aging. However, some limitations should be acknowledged. First, the cross-sectional design limits causal inference, and longitudinal studies are needed for confirmation. Second, dietary intake was assessed via a single 24-hour recall, which may not accurately capture long-term habits. Third, residual confounding from factors such as physical activity, sleep, or inflammatory markers cannot be ruled out. Finally, mediation analysis reveals statistical pathways but does not confirm biological causality, which requires further experimental validation.

In conclusion, our findings support the protective role of high diet quality in mitigating biological aging and identify WHtR and BRI as key mediators in this relationship. These insights provide a theoretical basis and practical direction for developing integrated strategies that combine nutritional and body shape management to promote healthy aging.

## 5. Conclusion

In conclusion, our study provides consistent evidence that higher diet quality is protective against biological aging, with this relationship being significantly mediated by central adiposity. By identifying WHtR and BRI as more informative mediators than BMI, our findings underscore the limitations of weight-centered screening approaches and emphasize the importance of abdominal fat distribution. These insights support the development of integrated public health strategies that combine nutritional improvement with targeted management of central adiposity to promote healthy longevity across diverse populations.

## Acknowledgments

The author would like to thank the participants and investigators of the National Health and Nutrition Examination Survey (NHANES) for providing the publicly available data used in this study. Their contributions made this research possible.

## Author contributions

**Data curation:** Yingying Qian.

**Methodology:** Yingying Qian.

**Software:** Yingying Qian.

**Supervision:** Yingying Qian.

**Validation:** Yingying Qian.

**Writing – original draft:** Yingying Qian.

**Writing – review & editing:** Yingying Qian.










