# Supplementary material for: Comparative mediation roles of body shape indices in the association between Healthy Eating Index-2015 and phenotypic age acceleration: A cross-sectional study based on NHANES 2005–2010
Source: Medicine (Baltimore). 2026 Sep 18;105(38):e50734. doi: 10.1097/MD.0000000000050734 (PMC13592949; doi:10.1097/MD.0000000000050734)
Supplement: Supplementary file 1 [file medi-105-e50734-s001.docx]

**Supplementary tables**

| **Table S1. Missing data rates for covariates included in the analysis** | | |
| --- | --- | --- |
| Variable | Missing Count | Missing Rate (%) |
| INDFMPIR | 885 | 35.06 |
| ALQ101 | 566 | 22.42 |
| BPQ020 | 20 | 0.79 |
| MCQ220 | 15 | 0.59 |
| DMDEDUC2 | 13 | 0.52 |
| HEI-2015_SATFAT | 10 | 0.40 |
| DIQ010 | 10 | 0.40 |
| DMDMARTL | 5 | 0.20 |
| SMQ020 | 2 | 0.08 |
| HEI-2015_SODIUM | 2 | 0.08 |
| **Note:** Missing data rates were calculated based on the total analytical sample. | | |
