# Supplementary material for: Comparative mediation roles of body shape indices in the association between Healthy Eating Index-2015 and phenotypic age acceleration: A cross-sectional study based on NHANES 2005–2010
Source: Medicine (Baltimore). 2026 Sep 18;105(38):e50734. doi: 10.1097/MD.0000000000050734 (PMC13592949; doi:10.1097/MD.0000000000050734)
Supplement: Supplementary file 2 [file medi-105-e50734-s002.docx]

| **Table S5. Comparison of HEI-2015 component scores between participants with and without phenotypic age acceleration** | | | |
| --- | --- | --- | --- |
| **HEI-2015 component scores** | Delayed  N = 10489 (87%) | Accelerated  N = 2022 (13%) | **p-value** |
| Total HEI-2015 score | 51 (43, 60) | 48 (41, 56) | <0.001 |
| HEI-2015_TOTALFRT | 2.47 (0.47, 3.91) | 2.07 (0.20, 3.56) | <0.001 |
| HEI-2015_FRT | 2.50 (0.10, 4.20) | 1.68 (0.00, 3.05) | <0.001 |
| HEI-2015_VEG | 3.27 (2.26, 4.25) | 3.09 (2.09, 4.09) | 0.008 |
| HEI-2015_GREENNBEAN | 0.87 (0.00, 2.50) | 0.00 (0.00, 2.50) | 0.004 |
| HEI-2015_TOTALPRO | 4.65 (3.71, 5.00) | 4.68 (3.71, 5.00) | 0.5 |
| HEI-2015_SEAPLANTPRO | 2.50 (0.39, 3.59) | 2.25 (0.00, 2.61) | <0.001 |
| HEI-2015_WHOLEGRAIN | 1.73 (0.00, 4.37) | 1.23 (0.00, 3.74) | <0.001 |
| HEI-2015_DAIRY | 5.29 (3.07, 7.51) | 5.02 (2.80, 7.31) | 0.017 |
| HEI-2015_FATTYACID | 4.98 (2.60, 6.97) | 4.54 (2.28, 6.63) | <0.001 |
| HEI-2015_REFINEDGRAIN | 6.35 (4.20, 8.68) | 6.51 (4.26, 8.91) | 0.4 |
| HEI-2015_SODIUM | 4.31 (2.02, 6.45) | 4.20 (2.08, 6.37) | 0.9 |
| HEI-2015_ADDEDSUGAR | 7.33 (4.73, 9.23) | 6.88 (4.18, 9.17) | 0.007 |
| HEI-2015_SATFAT | 6.33 (4.06, 8.38) | 5.73 (3.42, 8.01) | <0.001 |
| **Note:** Accelerated aging was defined as phenotypic age acceleration (PhenoAgeAccel) ≥ 0, and delayed aging was defined as PhenoAgeAccel < 0. Values are presented as weighted medians with interquartile ranges. Differences between groups were assessed using the Wilcoxon rank-sum test due to the non-normal distribution of HEI-2015 component scores.  **Abbreviations:** HEI-2015, Healthy Eating Index–2015; FRT, fruit; VEG, vegetables; PRO, protein foods; SATFAT, saturated fat. | | | |
