# Supplementary material for: Comparative mediation roles of body shape indices in the association between Healthy Eating Index-2015 and phenotypic age acceleration: A cross-sectional study based on NHANES 2005–2010
Source: Medicine (Baltimore). 2026 Sep 18;105(38):e50734. doi: 10.1097/MD.0000000000050734 (PMC13592949; doi:10.1097/MD.0000000000050734)
Supplement: Supplementary file 3 [file medi-105-e50734-s003.docx]

| **Table S3. Threshold effect analysis of the association between HEI-2015 score and phenotypic age acceleration using segmented regression** | | |
| --- | --- | --- |
| Outcome | effect | *P* |
|  |  |  |
| Model 1 Fitting model by standard linear regression | 0.98 (0.98,0.99) | **<0.001** |
| Model 2 Fitting model by two-piecewise linear regression |  |  |
| Inflection point | 60.29 |  |
| <60.29 | 0.99 (0.98, 0.99) | **<0.001** |
| ≥60.29 | 0.98 (0.96 , 1.00) | **0.021** |
| P for likelihood test |  | 0.490 |
| **Note:** Segmented (two-piecewise) linear regression models were used to examine potential threshold effects in the association between HEI-2015 score and phenotypic age acceleration (PhenoAgeAccel). Regression coefficients (β) and 95% confidence intervals (CIs) were estimated. The likelihood ratio test comparing the segmented model with a linear model was not statistically significant (P = 0.490), indicating no evidence of a threshold effect and supporting an approximately linear association across the observed range of HEI-2015 scores.  **Abbreviations:** HEI-2015, Healthy Eating Index–2015; PhenoAgeAccel, phenotypic age acceleration; CI, confidence interval. | | |
