# Supplementary material for: Comparative mediation roles of body shape indices in the association between Healthy Eating Index-2015 and phenotypic age acceleration: A cross-sectional study based on NHANES 2005–2010
Source: Medicine (Baltimore). 2026 Sep 18;105(38):e50734. doi: 10.1097/MD.0000000000050734 (PMC13592949; doi:10.1097/MD.0000000000050734)
Supplement: Supplementary file 4 [file medi-105-e50734-s004.docx]

| **Table S2. Association between HEI-2015 and phenotypic age acceleration: multivariable linear regression models** | | | | | | | | |
| --- | --- | --- | --- | --- | --- | --- | --- | --- |
| Variable | Model1 | |  | Model2 | |  | Model3 | |
|  | β (95%CI) | *P* |  | β (95%CI) | *P* |  | β (95%CI) | *P* |
| HEI-2015  (per 1-point increase) | -0.08  (-0.09 , -0.07) | **<0.001** |  | -0.07  (-0.08 ,-0.06) | **<0.001** |  | -0.06  (-0.07 , -0.05) | **<0.001** |
| **Note:** Regression coefficients (β) and 95% confidence intervals (CIs) were estimated using multivariable linear regression models, with phenotypic age acceleration (PhenoAgeAccel) as the outcome. PhenoAgeAccel represents the difference between phenotypic age and chronological age.  Model 1: Unadjusted.  Model 2: Adjusted for age, sex, race/ethnicity, education level, income-to-poverty ratio, and marital status.  Model 3: Further adjusted for smoking status, alcohol use, cancer history, hypertension, and total energy intake (kcal).  **Abbreviations:** HEI-2015, Healthy Eating Index–2015; CI, confidence interval. | | | | | | | | |
