# Supplementary material for: Comparative mediation roles of body shape indices in the association between Healthy Eating Index-2015 and phenotypic age acceleration: A cross-sectional study based on NHANES 2005–2010
Source: Medicine (Baltimore). 2026 Sep 18;105(38):e50734. doi: 10.1097/MD.0000000000050734 (PMC13592949; doi:10.1097/MD.0000000000050734)
Supplement: Supplementary file 5 [file medi-105-e50734-s005.docx]

| ****Table S4. Associations of HEI-2015 with adiposity indices and their associations with phenotypic age acceleration**** | | | | |
| --- | --- | --- | --- | --- |
| Adiposity index | HEI-2015 → Adiposity index  β (95% CI) | p value | Adiposity index → PhenoAgeAccel  β (95% CI) | *p*-value |
| BMI | -0.054 (-0.064 , -0.044) | <0.001 | 0.282 (0.264 , 0.300) | <0.001 |
| BRI | -0.020 (-0.023 , -0.017) | <0.001 | 0.956 (0.902 , 1.011) | <0.001 |
| ABSI | -0.000 (-0.000 , -0.000) | <0.001 | 222.710 (195.768 , 249.652) | <0.001 |
| WHtR | -0.001 (-0.001 , -0.001) | <0.001 | 22.757 (21.471 , 24.043) | <0.001 |
| **Note:** All regression coefficients (β) were derived from multivariable linear regression models.  Models were adjusted for age, sex, race/ethnicity, education level, income-to-poverty ratio (PIR), marital status, smoking status, alcohol use, history of cancer, hypertension, and total energy intake.  ABSI was calculated using the standard formula based on waist circumference, body mass index, and height. Larger β coefficients reflect the unit scale of ABSI rather than a large biological effect.  **Abbreviations:** HEI-2015, Healthy Eating Index–2015; PhenoAgeAccel, phenotypic age acceleration; BMI, body mass index; BRI, body roundness index; WHtR, waist-to-height ratio; ABSI, a body shape index; CI, confidence interval. | | | | |
